# The Essentials of Histology, Descriptive and Practical

**Published:** 1911-01

**Authors:** 


**The Essentials of Histology, Descriptive and Practical.** For the use of students. By **Edward A. Schafer,** F.R.S., Professor of Physiology in the University of Edinburgh. Eighth edition. Octavo, 571 pages, with 645 illustrations. Lea & Febiger, Publishers, Philadelphia and New York. 1910. (Cloth, $3.50, net.)

This book is intended to serve as an elementary textbook of histology. It comprises all the essential facts of the science expressed as briefly as can be done without abridgement of clearness. For the convenience of the student the book is divided into fifty lessons, each of which are supposed to occupy from one to three hours. This, the eighth edition, is somewhat larger than the one before it, the increase being mainly due to additional illustrations. Many of these are photographs of microscopic preparations, and are excellent productions. A knowledge of all this book contains will be sufficient for the needs of the average student.

